# The drive to generate multiple forms of oncogenic cyclin D1 transcripts in mantle cell lymphoma

**DOI:** 10.1186/s40364-017-0094-7

**Published:** 2017-05-08

**Authors:** Chioniso Patience Masamha

**Affiliations:** Department of Pharmaceutical Sciences, College of Pharmacy and Health Sciences, Butler University, 4600 Sunset Avenue, Indianapolis, IN 46208 USA

**Keywords:** Mantle cell lymphoma, Alternative polyadenylation, 3′UTR shortening, Cyclin D1, Polyadenylation signal

## Abstract

Alternative polyadenylation is a rapidly emerging form of gene regulation, which in its simplest form, enables the generation of mRNA transcripts that code for the same protein but have 3′UTRs of different lengths and regulatory content. For oncogenes, shorter 3′UTRs would be preferred as a mechanism to evade miRNA regulation. The shortening of the 3′UTR of cyclin D1 in mantle cell lymphoma offers provocative insights into this process. Patient samples have revealed that 3′UTR shortening may occur due to mutations, or translocations that result in the generation of a chimeric 3′UTR. The truncated cyclin D1 3′UTRs resulting from alternative polyadenylation, use a premature canonical polyadenylation signal close to the stop codon that was generated either as a result of mutations or provided by another gene in the chimeric 3′UTR. The sequence of the polyadenylation signal in mantle cell lymphoma appears to be critical for 3′end formation of the cyclin D1 transcript. Shortening the 3′UTR allows cyclin D1 to potentially evade regulation by over 80 miRNAs that are predicted to bind to its full length 3′UTR.

## Background

The G1-S phase cell cycle regulatory oncogene cyclin D1 plays a major role in many cancers but appears to be the central driver of Mantle cell lymphoma (MCL) pathogenesis. MCL is a highly aggressive B-cell lymphoma which is considered clinically incurable upon disease relapse. The initiating lesion for MCL tumorigenesis is believed to be the aberrant expression of cyclin D1 (*CCND1*). Accidents that occur during recombination of the V(D)J segments of pre-B stage lymphocytes during differentiation give rise to the characteristic t(11;14)(q13;q32) translocation event that juxtaposes the *CCND1* allele downstream of the IgH intronic regulatory enhancer (Eμ) element [1]. The subsequent recruitment of RNA polymerase II, nucleolin and other factors facilitate the transcriptional activation of the *CCND1* promoter which is silent in normal B-lymphocytes [2, 3]. Current diagnoses of MCL include analysis of cyclin D1 translocation/overexpression. However, the transcriptional activation of the *CCND1* promoter seems to be just the beginning of how cyclin D1 protein expression is aberrantly regulated in MCL. There are extra mechanisms that further serve to enhance the stability of the transcribed *CCND1* mRNA, hence abnormally sustaining its translation.

### The importance of cyclin D1 mRNA in mantle cell lymphoma

The nature of the *CCND1* mRNA transcript plays a major role in predicting survival of MCL patients. MCL patients who express the full *CCND1* transcript will on average survive ~2 years longer than patients who express a *CCND1* transcript with a truncated 3′untranslated region (3′UTR), while retaining the same protein coding sequence [4]. Using StarBase analysis which links miRNA-mRNA data with CLIP-Sequencing data, we found that the full length cyclin D1 3′UTR transcript can be potentially bound by and regulated by 86 miRNAs [5]. A different analysis using miRanda predicted 58 miRNAs with the potential to bind *CCND1*’s 3′UTR [6]. Furthermore, a genome-wide profile study of cyclin D1-positive MCL patient tissues showed significant up-regulation of miR-19a and miR-19b [7] which were among the miRNAs we identified using StarBase. We also verified the role of miR-19a in regulating *CCND1* mRNA levels using a miRNA mimic [5]. Given the high number of potential miRNA binding sites, it is not surprising that *CCND1* may try to evade miRNA by altering the length of its 3′UTR through alternative polyadenylation. Alternative polyadenylation is emerging as a widespread and important form of gene regulation that involves 3′end formation which, in its simplest form, involves changes within the same terminal exon.

### Sequences and factors involved in 3′end formation

The processing of the 3′end of a transcript is regulated by several *cis* regulatory elements within the pre-mRNA. The 3 core elements include the polyadenylation signal (PAS), which is followed by the cleavage site, and a GU/U rich downstream sequence element (DSE) [8]. In addition, there are 2 auxiliary sequences which consist of an upstream sequence element (USE) and a poorly understood G-rich downstream element [8, 9]. These 3′UTR *cis*-elements are important because they are the binding sites for the 3′ end processing factors, which make up the cleavage and polyadenylation complex. The key subunits of the complex are made up of 4 multi-protein components: the cleavage and polyadenylation specificity factors (CPSFs), the cleavage stimulation factors (CstFs), and the mammalian cleavage factors (CFIm and CFIIm). During cleavage and polyadenylation, Wdr33 and the CPSF30 subunit directly bind the PAS hexamer. The CstF64 subunit binds to the G/GU rich downstream sequence element on the pre-mRNA. The binding of these factors to the pre-mRNA facilitates the recruitment of the remainder of the 3′end processing machinery. As a result, the pre-mRNA cleavage site is brought into proximity with the endoribonuclease CPSF73, which then cleaves the pre-mRNA. Then poly (A) polymerase adds the poly (A) tail. [8, 10–12]. Some of the members of the 3′end processing complex bind to RNA polymerase II, and are carried along during transcription. Hence although 3′end formation contains distinct biochemical steps, it occurs simultaneously with transcription and splicing, and 3′end formation is closely coupled to transcription termination [11, 13].

### The role of the polyadenylation signal in 3′end processing

**Fig. 1:**
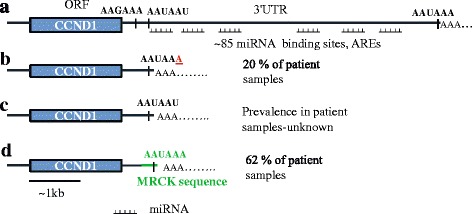
The diversity of cyclin D1 transcripts in mantle cell lymphoma. This work is based on a previous publication [5]. **a** A schematic showing the location and sequences of all the potential polyadenylation signals (PAS) in full length *CCND1* mRNA with the canonical polyadenylation signal (AAUAAA) at the most distal terminus. **b** Schematic of the PAS used in the alternative polyadenylation of the *CCND1* in some MCL patients. The *underlined letter* (in *red*) is mutated in these patients to generate a canonical PAS. **c** The unaltered PAS which was identified in the Jeko-1 MCL cell line is shown and it is downstream of the other proximal hexamer. **d** A diagrammatic representation of the *CCND1/MRCK* fusion chimera showing the location of the MRCK sequence (in *green*) in the chimera and the PAS it contains

The importance of each *cis* element in 3′end formation varies from one pre-mRNA to the next. The major determinant of 3′end formation is the polyadenylation signal (PAS); and cleavage of the pre-mRNA occurs ~15 nucleotides downstream of the PAS. In nearly 70% of the annotated genome, the PAS consists of the canonical hexamer A(A/U)UAAA while the rest of the pre-mRNAs have sequences that often contain one or more nucleotide substitutions [8]. The prevailing theory is that the canonical signal is processed more efficiently than other variations. Interestingly, the full length *CCND1* 3′UTR contains several potential PASs where alternative polyadenylation can potentially occur (Fig. 1a). The canonical PAS (AAUAAA) is more distal to the open reading frame, whereas the two other potential non-canonical PASs (AAGAAA and AAUAAU) are located closer to the stop codon. In cases where more than one PAS exists, it is hypothesized that the most distal PAS typically contains the canonical hexamer, and ‘weaker’ variants of the sequence are typically located in the region more proximal to the open reading frame end [14]. The implication of this trend is that the default choice is to utilize the more distal canonical PAS. To underscore the role of the PAS, mutations that affect the hexamer have been reported to play a role in altered poly(A)site selection in various diseases including thrombophilia and thalassemia [15].

### The polyadenylation signals used determines the length of the cyclin D1 transcript

**Fig. 2:**
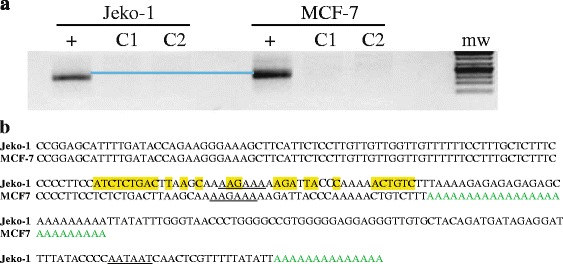
Mantle cell lymphoma uses a different polyadenylation sequence than other cancer cells. **a** PCR products derived from 3′RACE of mRNA isolated from MCF7 and Jeko-1. C1 represents minus reverse transcriptase controls and C2 are controls minus the oligo(dT25)T7 primers. The *blue line* was inserted to highlight the slight difference in the size of the two 3′UTR transcripts. The method for 3′RACE was previously described [5]. **b** A partial sequence of the 3′UTR cDNA sequence derived from MCF7 compared to that of Jeko-1 showing the polyA tail (in *green*) for each transcript together with the PAS (*underlined*). Highlighted in *yellow* are differences in the nucleotide sequences between the two sequences, upstream of the first cleavage and polyadenylation site

A small number of MCL patients had mutations that resulted in the generation of an optimal polyadenylation sequence (AAUAAA) in *CCND1* from the AAUAAU hexamer (Fig. 1b). These patients had highly proliferative tumors that were associated with poor prognosis [4]. In one out of three MCL cell lines, we found that in Jeko-1, the non-canonical proximal PAS (AAUAAU) was used without any mutations, generating a 3′UTR of 318 nucleotides through alternative polyadenylation (Fig. 1c) [5]. Interestingly, when we performed 3′RACE on the breast cancer cell line, MCF7 we found that this cell line generates a transcript with a slightly shorter 3′UTR compared to alternatively polyadenylated Jeko-1 (Fig. 2a). Sanger sequencing revealed that the breast cell line uses the alternative non-canonical proximal AAGAAA hexamer, which is upstream of the one used by Jeko-1 (Fig. 2b). However, although using alternative polyadenylation in the presence or absence of mutations to the PAS to generate a truncated *CCND1* 3′UTR does occur in MCL, it is not very prevalent. A significant number of MCL patients were previously reported to have uncharacterized genomic translocations that resulted in the chromosomal deletion of the 3′UTR [4]. We identified a novel truncated *CCND1* 3′UTR that uses a canonical, optimal PAS (AAUAAA) derived from another gene’s intronic sequence (Fig. 1d) [5].

Using a combination of different techniques together with Sanger sequencing we found that in two out of three MCL cell lines as well as 8/13 MCL patient samples the full open reading frame of *CCND1* and a small segment of the adjacent 3′UTR was fused to a stretch of nucleotides derived from myotonic dystrophy kinase-related Cdc42-binding kinase (*MRCK*). This generates a chimeric 3′UTR, consisting of 361 nucleotides from both *CCND1* and *MRCK*. The attached *MRCK* sequence contains a consensus PAS (AAUAAA) that is optimal for 3′end formation. Further studies showed that the sequence was the reverse complement of a portion of the MRCKs intron one region. Our results using a luciferase reporter containing this chimeric 3′UTR sequence showed that the *CCND1/MRCK* fusion gene is recalcitrant to regulation by three separate miRNAs [5].

## Conclusion

There appears to be strong drive to express *CCND1* in MCL. In addition to abnormal transactivation of the promoter, truncations of the 3′UTR to generate transcripts that are recalcitrant to miRNA regulation result in higher cyclin D1 protein expression. We described a novel *CCND1/MRCK* fusion gene in MCL. In the *CCND1/MRCK* chimera, the truncated 3′UTR contains sequences from both genes, with the *MRCK* portion providing a canonical PAS to facilitate 3′end formation. This opens up a new potential avenue to generate therapeutics that will differentially target the *MRCK* sequences and are hence more specific for MCL without impacting normal *CCND1* expression in other human tissues.

## Abbreviations

3′RACE: 3′rapid amplification of cDNA ends
3′UTR: 3′untranslated region
CCND1: cyclin D1
Eμ: IgH intronic regulatory enhancer (Eμ) element
MCL: Mantle cell lymphoma
MRCK: Myotonic dystrophy kinase-related Cdc42-binding kinase
PAS: Polyadenylation signal
